# RiGoR: reporting guidelines to address common sources of bias in risk model development

**DOI:** 10.1186/s40364-014-0027-7

**Published:** 2015-01-24

**Authors:** Kathleen F Kerr, Allison Meisner, Heather Thiessen-Philbrook, Steven G Coca, Chirag R Parikh

**Affiliations:** Department of Biostatistics, University of Washington, Box 357232, Seattle, WA 98195 USA; Kidney Clinical Research Unit Room ELL-101, Westminster Tower London Health Sciences Centre, 800 Commissioners Road East, London, ON N6C 6B5 Canada; Icahn School of Medicine at Mount Sinai, One Gustave L. Levy Place, Box 1243, New York, NY 10029 USA; Yale University School of Medicine Program of Applied Translational Research, Temple Street, Suite 6C, New Haven, CT 06510 USA

**Keywords:** Risk prediction, Reporting standards, Research design, Statistical bias

## Abstract

Reviewing the literature in many fields on proposed risk models reveals problems with the way many risk models are developed. Furthermore, papers reporting new risk models do not always provide sufficient information to allow readers to assess the merits of the model. In this review, we discuss sources of bias that can arise in risk model development. We focus on two biases that can be introduced during data analysis. These two sources of bias are sometimes conflated in the literature and we recommend the terms *resubstitution bias* and *model-selection bias* to delineate them. We also propose the RiGoR reporting standard to improve transparency and clarity of published papers proposing new risk models.

## Introduction

There is currently broad interest in developing risk prediction models in medicine. However, recent reviews in a variety of fields have described a substantial number of flaws in the way risk models are developed and/or deficiencies in the way the work is reported [1-6]. An extensive review that spanned many fields of application found the vast majority of papers reporting risk models omitted important details such as: the extent and handling of missing data; key information on the study population; and the precise definition of the outcome or event of interest [6]. An evaluation of model calibration was typically absent. Additional issues include a tendency for models to be favorably evaluated when the model’s developers are involved in validating the model [4].

Research reports related to risk prediction sometimes refer to “optimistic bias” or “optimism bias” [4,7,8]. Unfortunately, these terms are used to refer to a variety of problems in risk model development or assessment. It would be useful to have clear, distinctive, and descriptive names for different sources of bias that can affect scientific results. The first goal of this review is to propose terminology for referring to two sources of bias that are common in developing risk models. Both biases can arise during data analysis, which makes them avoidable, at least in principle. The second goal of this paper is a proposal for a set of guidelines for reporting proposed new risk models. The guidelines should help readers evaluate the merits of new risk models and understand whether developers were attentive to avoiding common sources of bias.

## Review

### Common sources of bias in risk model development

Currently, two sources of bias that arise in developing risk prediction models from combinations of biomarkers and/or clinical variables are both called “optimistic bias.” We propose the terms “resubstitution bias” and “model-selection bias” as more precise and descriptive terms than “optimistic bias.” A predictive model will tend to perform better on the data that were used to fit or “train” the model than on new data. Resubstitution bias arises when the data that are used to fit a predictive model are used a second time to assess the performance of the model. Re-using the data in this way has been called resubstitution [9-15], so it is a modest extension to refer to the resulting bias as resubstitution bias. Since the ultimate goal of a risk prediction model is to estimate risks on new individuals, assessing model performance via resubstitution does not provided an unbiased or “honest” estimate of the model’s predictive capacity.

Model-selection bias arises when many models are assessed, and the best performing model is reported. This optimistic bias persists even if analysts have corrected for resubstitution bias in assessing the model. Occasionally, investigators have a single, pre-specified model that they fit with data. In this case, the resulting model is susceptible to resubstitution bias but not to model-selection bias. More typically, however, data analysts have a set of candidate predictors to choose from, which translates to a set of possible models. For example, if there are k candidate predictors and an analyst limits the set of possible models to linear logistic models, then the number of possible models is 2^k^-1. For 20 candidate predictors this is over 1 million models, and we expect some of these to perform well by chance. Naïve assessment of the best-performing model is likely optimistic because this model is chosen because it performed best on the available data [16-19]. “Model-selection bias” refers to this particular source of optimism.

Although resubstitution bias and model-selection bias are well-known phenomena among methodologists and many data analysts [7,10,20], there is no standard terminology for referring to these sources of bias. We find the term “optimistic bias” inadequate for several reasons. “Optimistic bias” describes the direction of the bias and not the source of the bias, so it is insufficiently descriptive. By referring to multiple sources of bias with the same phrase researchers might claim to have addressed “optimistic bias” in developing a predictive model [21], when in fact they have only addressed one source of bias. Finally, in addition to resubstitution and model-selection there are additional phenomena referred to as “optimistic bias” [8], including the observation in psychology that people often underestimate personal risks [22].

We emphasize that resubstitution bias and model-selection bias are well-known among methodologists, and our modest contribution is our proposal for standard terms to refer to these issues. These terms have appeared occasionally (and rarely) in the literature [17,23,24] but are not in widespread use. Previously proposed terminology is “parameter uncertainty” and “model uncertainty [20], where “model uncertainty” is said to lead to “selection bias.” However, this terminology is not standard and we find it less descriptive than the terms we propose. Furthermore, “selection bias” has an established meaning in epidemiology, where it refers to non-representative selection of study subjects.

Methodology for estimating the performance of a risk model that is not optimistically biased from resubstitution includes bootstrapping techniques, cross-validation, and using independent datasets for model development and validation [7,25]. Bootstrapping and cross-validation are computationally intensive, and employing them can surpass the abilities of some data analysts or software packages. Moreover, there are different varieties of bootstrapping and cross-validation and a lack of consensus on the best procedure. A recent investigation [21] provides some much-needed guidance on the relative merits of different procedures for estimating the area under the ROC curve (AUC or “C statistic”) without resubstitution bias. Using independent datasets for model development and validation is computationally simpler, and provides stronger evidence in favor of a reported risk model if the validation dataset is from a separate study (“external validation”). More commonly, however, a validation dataset is created by data splitting – randomly partitioning the available data into a “training” dataset and a “test” or validation dataset. This strategy offers simplicity and flexibility in data analysis, but is criticized for its statistical inefficiency [7] because only part of the data inform development of the risk model. With data splitting there is an inherent tension between the amount of data allotted to the training dataset for developing the risk model, and the amount of data allotted to the test dataset for evaluating the risk model [26]. If the training dataset is too small a good risk model might not be found. On the other hand, if the test dataset is too small then estimates of model performance, while unbiased, are highly variable, making promising results less compelling. An advantage of having an independent validation dataset is that both resubstitution and model-selection bias are accounted for as long as the validation dataset is not used in any stage of model development, including variable selection.

Model-selection bias tends to be more difficult to address without an independent validation dataset. In principle, model-selection can be incorporated into a bootstrapping or cross-validation procedure, but this requires the use of an automated model-building process and further increases the computational complexity of using these methods.

### Reporting standards

There have been several efforts to develop standards and guidelines for reporting various types of scientific studies. As summarized by McShane and colleagues [27], quality study reporting “cannot transform a poorly designed or analyzed study into a good one, but it can help to identify the poor studies.” Quality reporting is an important first step in improving the overall quality of risk model development work [4,6]. Reporting standards can additionally help guide researchers toward best practices. Table 1 presents our proposed RiGoR (Reporting Guidelines for Risk Models) Standards for reporting risk model development.

**Table 1:** **RiGoR: reporting guidelines for risk models**

			**Similar items**		
**Section and topic**	**Item**		**STARD**	**REMARK**	**GRIPS**
Title/Abstract/Keywords	1	Identify the article as reporting the development of a risk model combining multiple predictors (MeSH “Risk”, possibly “risk factor” and/or “biomarker”)	1	1	1
Introduction	2	Identify the overarching goal – why would an effective risk model be valuable to clinical care, public health, or research?	2	20	2
Methods					
Participants	3	Describe the study subjects: The inclusion and exclusion criteria (and resulting sample sizes), setting and locations where the data were collected. Descriptive statistics should include variable ranges.	3	2	4,5,14
	4a	Describe participant recruitment.	4		5
	4b	Report when study was done, including beginning and ending dates of recruitment.	14	6	4
	5	Describe the study design. Was this a cohort study? A case–control study? Note: matched case control studies are generally not suitable for risk model development unless special methods and external data are used.	5	6	4
Biomarker Data	6	Describe data collection, including timing of specimen collection for biomarker measurement. Document where there was blinding to clinical outcomes.	8	4,5	
	7	Document technical specifications of biomarker materials and methods, including marker units. Describe possibility of batch effects, storage effects, number of freeze/thaw cycles, assay upper and lower limits. Document how biomarker values at the limits of detection were handled.		4,5	7
	8	For multi-center studies, document whether biomarker measurements can be considered comparable between study sites, or whether lab effect, platform differences, or variations in clinical practice may affect biomarker levels.	23		
Outcome variable	9	Describe how the outcome is defined (e.g., precise definition for disease diagnosis, or death from any cause vs. specific cause)		7	6
Statistical Methods	10	Document measures of model performance, e.g. AUC for risk models; sensitivity and specificity for a pre-selected risk threshold; report methods to quantify uncertainty (e.g., 95% confidence intervals via bootstrapping)	12		12
	11	Document how markers were used: transformations (e.g., log)? categorization of continuous variables? Other adjustments (e.g. kidney biomarkers adjusted for urine creatinine)?	9	11	8
	12a	List all variables initially considered as candidates		8	9
	12b	Describe variable selection: how were variables selected to include in the risk model or classifier? Pre-specified prior to any analysis of the data? Selected based on univariate analysis? An exhaustive search over a set of models? Stepwise procedure?		10	9
	12c	Describe how model-selection bias was addressed in assessing the performance of final reported model(s). If model-selection bias was not addressed, state this explicitly.			10
	13	Document methodology used to develop risk model or classifier: logistic regression? logic regression? relative risk regression?		10	
	14a	Document methodology to avoid or correct for resubsitution bias in measures of the performance of the final reported model(s).			10
	14b	If an independent validation “test” dataset was used, document that the test data were not used for any part of model development, including variable selection. Document that these data were accessed only when models were finalized. Report the number of models evaluated on the “test” data and how these were selected.			10
	14c	If cross-validation is used, state how final reported model is derived.			10
	15	For multi-center studies with the possibility of confounding by center, describe methods for adjusting or accounting for center effects.			
	16	Describe how indeterminate results and missing data were handled, or report that there were no indeterminate results or missing data.	22		11
	17	Describe methods for assessing model calibration.			
Results	18	Report clinical and demographic characteristics of the study population (e.g. age, sex, presenting symptoms, co-morbidity, current treatments, recruitment centers).	15	13	15
	19	Report final risk model or classifier			
	20	Report estimates of model performance with measures of uncertainty when possible (e.g., 95% confidence interval)	21		18,19
	21	Assess and report evidence of risk model calibration.			
Discussion	22	Discuss prospects of final risk model for satisfying the research goal	25		22, 23
	23	Discuss known and possible limitations to generalizability or applicability of risk model		19	21

Previously published reporting standards that are related to risk model development are STARD [28], GRIPS [29], and REMARK [27]. The STARD initiative [28] assembled a comprehensive set of standards “to improve the accuracy and completeness of reporting of studies of diagnostic accuracy, to allow readers to assess the potential for bias in the study (internal validity) and to evaluate its generalisability (external validity)” (http://www.stard-statement.org/). A primary result of the initiative is a 25 item checklist for articles reporting studies of diagnostic accuracy. The RiGoR guidelines are meant to emulate the contribution of STARD with a set of criteria tailored to the development of risk prediction instruments. The REMARK recommendations [27] were developed in the context of tumor markers with the potential to be used for prognosis. The focus of REMARK is markers for predicting time-to-event outcomes such as overall survival. In contrast, the focus of RiGoR is estimating patient risks of a binary outcome. The GRIPS statement [29] offers reporting standards focused on studies of risk prediction models that include genetic variants. The RiGoR guidelines are more general and more detailed.

In proposing the RiGoR standards, we both acknowledge and build upon the important previous efforts described above. For each RiGoR item, Table 1 notes similar STARD, REMARK, or GRIPS items. As Table 1 shows, most items are similar to criteria given in at least one of these previous reports. However, there are some notable exceptions. First, RiGoR includes a guideline that the calibration of a risk prediction model should be assessed and reported, as calibration is a necessary requirement for the validity of a model. While the importance of calibration is noted in many publications [6,30,31], it is not included in GRIPS. Second, our guidelines explicitly address resubstitution bias and model-selection bias, two common types of bias that can arise during risk model development.

There are items in the REMARK and GRIPS guidelines that are not included in RiGoR. In Appendix A we document our reasons for excluding these items.

## Conclusions

In Epidemiology, common pitfalls in study design and data analysis commonly acquire standard names. Some examples include *immortal time bias* in survival analysis [32] and *lead time bias* in the evaluation of diagnostic screening tools [33]. *Publication bias* is a widely recognized issue in the scientific literature [34]. The most helpful terminology is descriptive; helps codify important concepts; and aids scientific communication. We believe the terms “resubstitution bias” and “model-selection bias” accomplish these goals.

In this article we have reviewed and discussed resubstitution bias and model-selection bias. We do not mean to suggest that they are the only two sources of bias that can affect risk model development. However, we believe resubstitution bias and model-selection bias deserve special attention because they are common. Furthermore, they are biases that arise during data analysis, which means, at least in principle, that they should be avoidable with use of proper methods of data analysis.

Other types of bias can enter into a study at earlier stages. For example, selection bias can inflate the performance of a proposed risk model if the cases in the dataset tend to be more severe than the population of cases, or controls tends to be healthier than the population of controls. Having an objective way to define the population of interest and to define the event of interest is an important aspect of a quality study. The RiGoR standards are designed to ensure that these and other important aspects of study design, conduct, and data analysis are documented.

## Appendix A

We comment on items in the GRIPS and REMARK guidelines that do not appear in the RiGoR guidelines. Our purpose here is explain the rationale of the RiGoR guidelines and our comments should not be interpreted as criticisms of these important, previous efforts.

### GRIPS

3. “Specify the study objectives and state the specific model(s) that is/are investigated. State if the study concerned the development of the model(s), a validation effort, or both.”

The RiGoR guidelines apply to papers documenting the development of risk models.

13. “Describe all subgroups, interactions, and exploratory analyses that were examined.”

20. “Present results of any subgroup, interaction, or exploratory analyses, whenever pertinent.”

Item 13 is very broad, and item 20 is vague as to what constitutes “pertinent” results. In the RiGoR guidelines, we focus on sources of bias (e.g. model-selection bias and resubstitution bias) that are common in risk model development and provide specific guidelines to identify them.

17. “Report distributions of predicted risks and/or risk scores.”

Such distributions are one way to evaluate model performance. The RiGoR guidelines provide flexibility on metrics by which to evaluate risk models.

24. “State whether databases for the analyzed data, risk models, and/or protocols are or will become publicly available and if so, how they can be accessed.”

We did not include a similar item in the RiGoR guidelines because it is not crucial for assessing the study that was done.

25. “Give the source of funding and the role of the funders for the present study. State whether there are any conflicts of interest.”

We did not include an item like this in the RiGoR guidelines because it is not specific to risk model research.

### REMARK

3. “Describe treatments received and how chosen (e.g. randomized or rule-based).”

This item is specific to prognosis in cancer.

9. “Give rationale for sample size; if the study was designed to detect a specified effect size, give the target power and effect size.”

This guideline is more appropriate for studies of association.

12. “Describe the flow of patients through the study, including the number of patients included in each stage of the analysis (a diagram may be helpful) and reasons for dropout. Specifically, both overall and for each subgroup extensively examined report the numbers of patients and the number of events.”

This item is not widely applicable to risk model development.

14. “Show the relation of the marker to standard prognostic variables.”

15. “Present univariate analyses showing the relation between the marker and outcome, with the estimated effect (e.g. hazard ratio and survival probability.) Preferably provide similar analyses for all other variables being analyzed. For the effect of a tumor marker on a time-to-event outcome, a Kaplan-Meier plot is recommended.”

16. “For key multivariable analyses, report estimated effects (e.g. hazard ratio) with confidence intervals for the marker and, at least for the final model, all other variables in the model.”

17. “Among reported results, provide estimated effects with confidence intervals from an analysis in which the marker and standard prognostic variable are included, regardless of their statistical significance.”

Such information is tangential to the validity and utility of a risk model. Furthermore, in the context of extensive variable or model selection, estimated effects may be biased.

18. “If done, report results of further investigations, such as checking assumptions, sensitivity analyses, and internal validation.”

This guideline is somewhat broad and open-ended, so we chose not to include a similar item in the RiGoR guidelines.
